# Dynamics of CWD prion detection in feces and blood from naturally infected white-tailed deer

**DOI:** 10.1038/s41598-023-46929-9

**Published:** 2023-11-17

**Authors:** Francisca Bravo‐Risi, Paulina Soto, Rebeca Benavente, Tracy A. Nichols, Rodrigo Morales

**Affiliations:** 1 Department of Neurology, The University of Texas Health Science Center at Houston, 6431 Fannin St., Houston, TX 77030 USA; 2 Centro Integrativo de Biologia y Quimica Aplicada (CIBQA), Universidad Bernardo O’Higgins, Santiago, Chile; 3 Veterinary Services Cervid Health Program, United States Department of Agriculture, Animal and Plant Health Inspection Service, Fort Collins, CO USA

**Keywords:** Biological techniques, Biotechnology, Neuroscience, Biomarkers, Diseases, Pathogenesis

## Abstract

Chronic wasting disease (CWD) is a prion disease affecting cervids. Confirmatory testing of CWD is currently performed *postmortem* in obex and lymphoid tissues. Extensive evidence demonstrates the presence of infectious prions in feces of CWD-infected deer using in vitro prion-amplification techniques and bioassays. In experimental conditions, this has been achieved as soon as 6-month post-inoculation, suggesting this sample type is a candidate for *antemortem* diagnosis. In the present study, we optimized the detection of CWD-prions in fecal samples from naturally infected, pre-clinical white-tailed deer by comparing protocols aiming to concentrate CWD-prions with direct spiking of the sample into the PMCA reactions. Results of this screening were compared with similar analyses made in blood. Our data shows that CWD-prion detection in feces using PMCA is best in the absence of sample pre-treatments. We performed a screening of 169 fecal samples, detecting CWD-prions with diagnostic sensitivity and specificity of 54.81% and 98.46%, respectively. In addition, the PMCA seeding activity of 76 fecal samples was compared with that on blood of matched deer. Our findings, demonstrate that CWD-prions in feces and blood are increased at late pre-clinical stages, exhibiting similar detection in both sample types (> 90% sensitivity) when PrP96GG animals are tested. Our findings contribute to understand prion distribution across different biological samples and polymorphic variants in white-tailed deer. This information is also relevant for the current efforts to identify platforms to diagnose CWD.

## Introduction

Chronic Wasting Disease (CWD) is a prionopathy affecting free-ranging^1–6^ and farmed cervids^3,7–9^. The agent responsible for the transmission and pathological progression of CWD is a misfolded form of the prion protein (PrP^Sc^) that spreads by promoting conformational changes in the host cellular prion proteins (PrP^C^)^3,10–12^. The abnormal aggregation and deposition of the prion misfolded fibrils occur mostly in the central nervous system^1,3,7,8,10^. Nonetheless, studies have demonstrated that CWD-prions are also present in extra neural tissues^13–28^. In addition, infectious prions are known to exist in excreta and bodily fluids of infected animals^29–36^, which may shed shortly after infection^37–40^ and contribute to environmental contamination. Considering the potential role of environmental fomites and humanmade materials in the transmission of CWD, the continuous shedding of prions is thought to play a relevant role in the expansion and recurrent outbreaks of this disease.

Confirmatory testing of CWD is currently performed by enzyme-linked immunosorbent assays (ELISAs) and/or immunohistochemistry (IHC) in biopsies of medial retropharyngeal lymph node (MRLPN) and obex tissues collected *postmortem*. In addition, recto-anal mucosa-associated lymphoid tissue (RAMALT) and MRLPN collected *antemortem* and tested by IHC are used to test white-tailed deer under specific situations^41^. A limitation in using *antemortem* diagnostic tests is that the collection of the samples is invasive, requiring the sedation of animals that in some cases die due to surgery-associated complications. In addition, these techniques require trained veterinarians and have limited sensitivity at early CWD post-exposure periods^15,31,42^.

The protein misfolded cyclic amplification (PMCA) and the real-time quaking-induced conversion (RT-QuIC) techniques are ultra-sensitive prion replication methods^43–48^ able to identify infectious prions in different sample types^15,19,31,32,48,49^. PMCA mimics the self-propagation of infectious prions in vitro through multiple sonication/incubation cycles, increasing the number of prion particles present in a given sample. The detection of proteinase K (PK)-resistant PrP^Sc^ by PMCA has been performed extensively in experimental and natural samples that may or may not harbor sub-infectious levels of prions^22,23,31,32,39,47,49–58^. Our group has explored the use of the PMCA technique as a tool to detect CWD-prions in multiple environmental and biological specimens (Refs.^23,56–59^ and unpublished). Importantly, this technique has been studied for its potential use in samples amenable with *antemortem* testing such as blood^56,57^, saliva^55^, feces^39,53^, and urine^32^.

As mentioned above, extensive evidence demonstrates the presence of infectious prions in excreta from deer^29,60^. Importantly, prion detection in these samples can be achieved long before the onset of clinical signs^29,38–40,61^. For example, PMCA analyses of feces from different cervid species (elk, mule deer, and white-tailed deer) demonstrated shedding as soon as 6-months post-infection^39^. This is relevant considering that PMCA detection identified the CWD status of mule deer using feces 3 months earlier compared to bioassays^29^. PMCA has also been reported to detect prions in feces 6-months earlier than RT-QuIC in white-tailed deer^40^. This and other data suggest that feces have the potential to be used in CWD diagnosis. Another relevant sample type that has been extensively explored for *antemortem* CWD testing is blood. CWD prionemia has been thoroughly demonstrated^56,57,62,63^. Our group has contributed to the optimization of CWD-prion detection in blood using specimens collected from naturally infected, non-clinical white-tailed deer^57^. Our published data demonstrate that PMCA is able to diagnose CWD in blood of white-tailed deer with a 100% specificity, and sensitivity that varies according to the progression of the disease and the PrP polymorphic variation at position 96^56,57^.

In the present study, we (i) evaluated three methods to detect PrP^Sc^ in white-tailed deer feces by PMCA; (ii) screened fecal samples from captive, naturally CWD-infected and pre-clinical white-tailed deer; and (iii) compared the CWD-prion detection using two extra neural samples (feces and blood) considering disease progression, genotyping variation, and sex. Our data provides valuable information regarding the use of blood and feces for CWD detection, and contributes to the understanding of prion accumulation in different sample types during the course of the incubation period.

## Materials and methods

### Fecal and blood samples collection and preparation

Fecal samples from 169 farmed white-tailed deer were opportunistically collected *postmortem* by USDA personnel. Fecal samples from each individual were collected with clean gloves, placed in 50 mL conical tubes and stored at − 80 °C until aliquoted into 1.5 mL microcentrifuges tubes. The samples were shipped to Dr. Morales’ laboratory at The University of Texas Health Science Center at Houston (UTHealth) and stored at − 20 °C until use. Fecal samples were thawed at room temperature and processed as described in Plummer et al.^39^ with minimal modifications. Briefly, 10% (w/v) feces homogenates were prepared in ultra-pure water containing a cocktail of protease inhibitors EDTA free (Roche, Basel, Switzerland) in MP Biomedical Lysing Matrix G 2 mL tubes containing a mix of glass beads and silicon carbide particles (MP Biomedicals, Irvine, CA, USA) and homogenized in a Bertin Precellys 24 dual homogenizer (MP Biomedicals, Irvine, CA, USA). Samples were placed in ice between homogenization cycles and stored at − 20 °C until being processed. Additionally, a subset of 76 white-tailed deer were blood-sampled. Blood specimens were collected *postmortem* from the jugular vein by USDA personnel and stored at -80 °C in tubes containing EDTA as described in Refs.^56,57^. PMCA results for these samples were previously described in these articles.

### Sodium phosphotungstic acid (NaPTA) treatment

The treatment was performed as described previously^39^ with slight modifications. One mL of feces homogenates was placed in 2 mL Lo-bind tubes (Eppendorf, Enfield, CT, USA) and supplemented with SDS (Sigma-Aldrich, Inc., St. Louis, MO, USA) to a final concentration of 1% (v/v). This mixture was vortexed, incubated in rotation for 1 h at room temperature, and then centrifuged for 1 h at 15,000×*g* and 10 °C. The supernatants were transferred to clean 1.5 mL Lo-bind tubes (Eppendorf, Enfield, CT, USA) and submitted to a new centrifugation step under the same conditions mentioned above. The supernatants were transferred to new 2 mL Lo-bind tubes (Eppendorf, Enfield, CT, USA) and 4% sarkosyl prepared in PBS was added in a 1:1 ratio. Afterward, a 4% sodium phosphotungstate hydrate solution prepared with ultra-pure water at pH 7.1 was added to the samples to reach a final concentration of 0.57%. The samples were incubated overnight at 37 °C in agitation (600 rpm) using an Eppendorf thermomixer (Eppendorf, Enfield, CT, USA). Products were again centrifuged for 30 min at room temperature and 15,000×*g*. The pellets were carefully rinsed with PMCA conversion buffer (1% triton X-100, and 150 mM NaCl prepared in PBS) containing a cocktail of protease inhibitors with EDTA (Roche, Basel, Switzerland) and centrifuged for additional 10 min at room temperature and 15,000×*g*. The final pellets were resuspended in PMCA substrate and submitted to in vitro prion replication.

### Sarkosyl treatment

Fecal and blood samples were treated with sarkosyl to concentrate their PrP^Sc^ content as previously described by our group^57^. Briefly, 200 µL of 10% (w/v) feces homogenates or blood were placed in 4.0 mL polycarbonate thick ultracentrifuge tubes (Beckman Coulter, Inc., Brea, CA, USA) containing 20% (w/v) sarkosyl (Thermo Fisher Scientific, Waltham, MA, USA) (prepared in PBS) in a 1:1 ratio. The mixtures were centrifuged for 1 h at 100,000×*g* and 4 °C and pellets were rinsed with 400 µL of 1× PBS containing a cocktail of EDTA-free protease inhibitors (Roche, Basel, Switzerland). Pellets were centrifuged again at 100,000×*g* for 30 min at 4 °C. Finally, pellets were resuspended in PMCA substrate and submitted to in vitro prion replication as described below.

### Protein misfolding cyclic amplification (PMCA)

The PMCA substrate was made using perfused brains from male and female homozygous tg1536 mice^64^ that were homogenized at 10% (w/v) in PMCA conversion buffer (150 mM NaCl and 1% Triton X-100 prepared in PBS) containing a cocktail of protease inhibitors with EDTA (Roche, Basel, Switzerland). Homogenates were centrifuged for 1 min at 805×*g* and 4 °C and supernatants were vortexed and aliquoted in 1.5 mL microcentrifuge tubes that were snap-frozen in liquid nitrogen and stored at − 80 °C. The PMCA substrate was supplemented with digitonin (Invitrogen, Carlsbad, CA, USA) and EDTA (Promega Corporation, Madison, WI, USA) at a final concentration of 0.025% and 6 mM, respectively, just prior use in the cyclic prion amplification reaction. Aliquots of 90 µL were transferred in 0.2 mL PCR tubes (Eppendorf, Enfield, CT, USA) containing three 3/32″ PTFE beads (Engineering Laboratories, Inc., Oackland, NJ, USA) and mixed with either 10 µL of 10% (w/v) feces homogenates or feces pellets. The mixtures were submitted to the first round of PMCA in a Qsonica sonicator Q700 with a microplate horn (QSONICA L.L.C, Newtown, CT, USA). The first PMCA round consisted of 144 cycles of incubation/sonication. Aliquots of these PMCA products (10 µL) were mixed with fresh PMCA substrate (90 µL) and subjected to two additional PMCA rounds of 96 cycles. Each PMCA cycle consisted of 29 min and 40 s of incubation, and 20 s of sonication at 37 °C. Each PMCA reaction set included serial dilutions of a CWD brain (10 µL) of known PMCA activity. As a negative control, four unseeded reactions were additionally included in each PMCA set. Each sample was blindly tested, in duplicate. Three different investigators participated in the screening process. The results were evaluated at the third round of PMCA. Fecal samples were considered CWD-positive if at least one of the replicates showed proteinase K (PK) resistant PrP^Sc^ signals in western blot (explained below).

### Proteinase K (PK) treatment

In order to detect PK resistant PrP^Sc^, PMCA products were treated with this protease (Sigma-Aldrich, Saint Louis, MO, USA) at a final concentration of 100 µg/mL and incubated at 37 °C for 90 min and shaking (450 rpm using an Eppendorf® thermomixer). The digestion reactions were stopped by adding NuPAGE LDS sample buffer (Invitrogen, Carlsbad, CA, USA) at a final concentration of 1× and heating the samples at 90 °C for 10 min.

### Western blot

To visualize PrP^Sc^ in PMCA products after PK treatment, samples were examined via western blot. First, samples were fractionated in NuPAGE 12% Bis–Tris gels (Invitrogen, Carlsbad, CA, USA). Electrophoresis was conducted at 80 V for 20 min and then 140 V for 100 min following the manufacturer’s recommendations. Proteins were transferred to a nitrocellulose membrane (GE Healthcare Amersham, Chicago, IL, USA) using cooled transfer buffer (0.025 M of tris base, 0.192 M of glycine, 10% (v/v) methanol) and blocked for 15 min at room temperature with 10% (w/v) non-fat milk solution. Membranes were probed using the monoclonal Bar224 antibody (Bertin Corp, Rockville, MD, USA) at a 1:10,000 dilution for 60 min at room temperature and incubated with secondary polyclonal Anti-Mouse IgG (whole molecule)-Peroxidase antibody produced in sheep (Sigma-Aldrich, Saint Louis, MO, USA) at a 1:3000 dilution for 60 min at room temperature. After incubation with each antibody, membranes were washed three times for 10 min at room temperature with washing buffer (1× PBS and 0.05% (v/v) Tween 20). The same buffer was used to prepare the milk and antibodies solutions. Membranes were developed using ECL (GE Healthcare Amersham, Chicago, IL, USA) following the manufacturer’s recommendations.

### Diagnostic sensitivity and specificity

Diagnostic sensitivity and specificity were calculated as in Parikh et al.^65^ using the following equations:

1 $$Diagnostic\, sensitivity=\frac{True \,positive }{(True \,positive+False\, negative) },$$

2 $$Diagnostic\, specificity=\frac{True\, negative }{(True\, negative+False \,positive) }.$$

### Ethical approval

Mice were bred, maintained, and sacrificed in approved facilities at UTHealth Houston. Euthanasia was performed by using CO_2_ inhalation following federal and local regulations, and approved by the Animal Welfare Committee of UTHealth at Houston (protocol AWC-22-0039). Animal manipulations described in this article followed the recommendations stated in the ARRIVE guidelines (https://arriveguidelines.org/). UTHealth investigators have USDA approval to receive and work with white-tailed deer-derived samples.

## Results

### Optimization of CWD-prion detection in fecal samples

**Figure 1:**
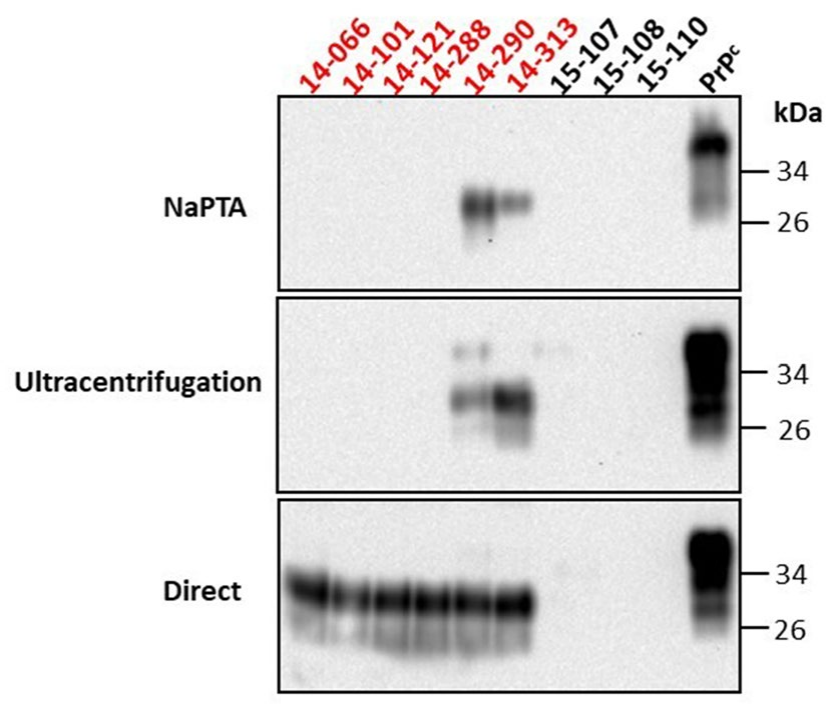
Pre-PMCA treatment on fecal samples results in variable detection outcomes. Fecal samples from CWD-detected (six, in red) and CWD-non-detect (three, in black) white-tailed deer by IHC (obex and retropharyngeal lymph node) were tested to detect seeding activity by PMCA. Analyses were performed comparing two pre-treatment methods (NaPTA and sarkosyl/ultracentrifugation) with no-treatment (direct spiking of feces homogenates in the PMCA reactions). PMCA products were treated with PK and evaluated as described in Materials and Methods. PrP^C^ represents non-PK-treated brain extracts from Tg1536^+/+^ mice used as a molecular weight migration control. The numbers at the right represent molecular weight markers.

Previous studies showed that NaPTA-based protocols are useful when studying samples harboring low levels of prions^14,15,37,39,50,66,67^. This has been particularly relevant for fecal samples analyzed with the RT-QuIC technique^16,40,61,68^. In addition, several reports described that in vitro PrP^Sc^ amplification efficiency might be affected by the sample matrix^35,52,55,57,67,69^ and NaPTA-based protocols may be useful in removing potential inhibitors, particularly the ones present in fecal samples^68^. In order to perform a CWD-prion screening from feces specimens collected from farmed/naturally-infected white-tailed deer, we evaluated two PrP^Sc^ enrichment methods: (i) NaPTA-based, and (ii) the sarkosyl/ultracentrifugation extensively used by our group^56–58^. Both methods were compared with (iii) direct spiking of feces homogenates to the PMCA substrate. For this optimization experiment, a set of nine fecal samples were selected: six specimens collected from late non-clinical white-tailed deer and tested as CWD-positive in blood by PMCA^57^, and three samples identified as CWD-non-detect (Table 1). The CWD-positive deer carried two 96G *PRNP* alleles. Importantly, PrP 96G alleles are suggested to favor peripheral PrP^Sc^ replication^14,15,25^. Our data shows that both NaPTA and sarkosyl/ultracentrifugation methods were able to identify prions in two samples only (Fig. 1, Table 1). Interestingly, the method without pre-treatments showed positive detection of PK-resistant PrP^Sc^ in all the fecal specimens collected from the CWD-positive animals (Fig. 1, Table 1).

**Table 1:** Demographics and PMCA results of the fecal samples used in the pre-treatment/optimization protocols.

Animal ID	IHC		PMCA (feces)			Genotype
	Obex	RPLN	NaPTA	Ultracentrifugation	Direct	
14–066	+	+	−	−	+	GG
14–101	+	+	−	−	+	GG
14–121	+	+	−	−	+	GG
14–288	+	+	−	−	+	GG
14–290	+	+	+	+	+	GG
14–313	+	+	+	+	+	GG
15–107	−	−	−	−	−	GG
15–108	−	−	−	−	−	N/D
15–110	−	−	−	−	−	N/D

*N/D* not determined.

### Screening of fecal samples from farmed white-tailed deer

Currently, official *postmortem*^41^ detection of CWD prions is performed by ELISA and/or IHC in obex and RPLN samples. However, several studies have shown that the accumulation of CWD prions in extra neural tissues and excreta occurs shortly after prion exposure, giving them potential diagnostic value^14,39,40,61,66^. In this study, we tested a set of 169 fecal samples from pre-clinical, CWD-infected white-tailed deer using the PMCA technique. From these samples, 104 specimens came from CWD-positive deer as tested by the conventional methods mentioned above. Importantly, the CWD status of the donor animals was blinded to the researchers performing the PMCA assay. CWD seeding activity was detected in 58 feces specimens from these CWD-positive animals, resulting in an overall diagnostic sensitivity of 54.81% (Table 2). The analysis of feces from CWD non-detect white-tailed deer provided a diagnostic specificity of 98.46% considering that a single sample from this group consistently presented with positive PMCA signals. This particular sample was evaluated by two different investigators, demonstrating seeding activity in 4/5 replicates. Considering this, results from this specific sample were considered as a false-positive.

**Table 2:** CWD diagnostic sensitivity and specificity in fecal samples from pre-clinical, farmed white-tailed deer.

True positive rate*	Diagnostic sensitivity	Diagnostic specificity
58/104	54.81%	98.46%

*True positive rate = n_PMCA positive_/n_IHC positive_.

### Effect of genotyping and disease progression on CWD-prion detection using fecal samples

The progression of prion disease is correlated with PrP^Sc^ detection in different tissues^14,66^, secreta^70^ and excreta^39,40^. To evaluate the detection of the infectious agent in fecal samples across the course of the prion incubation period in naturally infected white-tailed deer, we evaluated samples from 169 animals regardless of their PrP polymorphic identity. The classification of the disease progression was defined according to the presence or absence of CWD-staining in obex and/or retropharyngeal lymph node (RPLN) tissues by IHC testing, as in previous reports^56,57^. Animals were considered (i) early pre-clinical (n = 49) if they were IHC-positive in the RPLN only, or (ii) late pre-clinical (n = 55) if they were IHC-positive in the RPLN and obex. Deer were classified as CWD-non-detect (n = 65) if they were IHC-negative in both tissues. It is relevant to note that a single animal showed PrP^Sc^ aggregates in the obex, but not in the RPLN. This deer was classified as late pre-clinical, given that neuroinvasion in white-tailed deer occurs in late stages of the CWD incubation period. Our PMCA analyses show CWD-seeding activity in fecal samples from 46 (83.64%) deer at the late pre-clinical stage. At the early pre-clinical stage, just eleven (22.45%) were accurately identified (Table 3). This drop of prion detection in feces at late vs. early stages of the CWD incubation period is consistent with what has been described in other deer cohorts using RT-QuIC^16^.

**Table 3:** PMCA screening results in fecal samples from farmed white-tailed deer at early- and late-CWD pre-clinical stages.

Late pre-clinical true positive rate* (detection %)	Early pre-clinical true positive rate* (detection %)	Negative (detection %)
46/55* (83.64%)	11/49* (22.45%)	1/65 (1.54%)

*True positive rate = n_PMCA positive_/n_IHC positive_.

Subsequently, we explored the influence of polymorphic variation at codon 96 as this has been suggested as a modulator of peripheral prion replication and shedding^13,14,18,24,39,40,70^. A fraction (n = 123) of the total fecal samples were analyzed considering if the polymorphic identity at position 96 of PrP was available. In the PrP 96GG group, CWD-prion seeding activity was detected in 30 deer classified as late pre-clinical (93.75%). At the early pre-clinical stage, just 6 (26.09%) were accurately identified (Table 4). In the PrP 96GS late pre-clinical group, just 16 samples (65.57%) were detected as positive by PMCA. As expected, the diagnostic efficacy of PMCA decreased at the early pre-clinical stage as only 5 fecal samples (25.00%) were accurately diagnosed. In the PrP 96SS group, fecal samples from early pre-clinical animals only were collected. All samples from this group provided negative seeding activity in PMCA. In summary, these results clearly show that the presence of CWD-prions in feces is considerably more abundant in PrP 96GG white-tailed deer and at late pre-clinical stages.

**Table 4:** PMCA screening results in fecal samples from farmed white-tailed deer considering the progression of the pre-clinical stage and polymorphic variation at position 96 of the prion protein.

96GG (n = 58)			96GS (n = 50)			96SS (n = 15)		
Late pre-clinical true positive rate* (detection %)	Early pre-clinical true positive rate* (detection %)	Negative (detection %)	Late pre-clinical true positive rate* (detection %)	Early pre-clinical true positive rate* (detection %)	Negative (detection %)	Late pre-clinical true positive rate* (detection %)	Early pre-clinical true positive rate* (detection %)	Negative (detection %)
30/32* (93.75%)	6/23* (26.09%)	0/3 (0%)	16/23* (69.57%)	5/20* (25.00%)	0/7 (0%)	–	0/6* (0%)	1/9 (11.11%)

*True positive rate = n_PMCA positive_/n_IHC positive_.

### Detection of CWD-prions in fecal samples and blood from matched animals

In order to compare the CWD detection and prion content of feces and blood, we compared the seeding activity of sample pairs which were collected from the same deer. For that purpose, we analyzed matched samples from 76 deer with known PrP polymorphic variation at position 96 and IHC CWD status available. The results of blood used to compare feces samples results were previously reported by our group^56,57^. Our results demonstrate that the highest CWD-prion detection was obtained at the late pre-clinical phase in PrP 96GG animals. For this group, the CWD-prion seeding activity in fecal and blood specimen was equal to 92.59%. For PrP 96GS animals at the late pre-clinical stage, the detection of the infectious agent was higher in blood (70.00%) than in feces (60.00%) (Table 5). Unfortunately, no samples from PrP 96SS were available at late-preclinical stages, so analyses were not possible for this group of animals. This data confirms previous reports suggesting that the PrP 96G allele increases prion shedding.

**Table 5:** PMCA screening results in feces and blood from farmed white-tailed deer considering the progression of the pre-clinical stage and polymorphic variation at position 96 of the prion protein.

96GG (n = 47)						96GS (n = 23)						96SS (n = 6)					
Late pre-clinical true positive rate* (detection %)		Early pre-clinical true positive rate* (detection %)		Negative (detection %)		Late pre-clinical true positive rate* (detection %)		Early pre-clinical true positive rate* (detection %)		Negative detection %)		Late pre-clinical true positive rate* (detection %)		Early pre-clinical true positive rate* (detection %)		Negative (detection %)	
Feces	Blood	Feces	Blood	Feces	Blood	Feces	Blood	Feces	Blood	Feces	Blood	Feces	Blood	Feces	Blood	Feces	Blood
25/27* (92.59%)	25/27* (92.59%)	6/19* (31.58%)	10/19* (52.63%)	0/1 (0%)	0/1 (0%)	6/10* (60.00%)	7/10* (70.00%)	4/13* (30.77%)	7/13* (53.85%)	–	–	–	–	0/4* (0%)	0/4* (0%)	1/2 (50.00%)	0/2 (0%)

*True positive rate = n_PMCA positive_/n_IHC positive_.

At the early pre-clinical stage, animals harboring the PrP 96GG variant displayed an expected decrease in CWD detection by PMCA. This ranged from 31.58 to 52.63% in fecal and blood specimens, respectively (Table 5). A similar trend was observed in PrP 96GS deer, considering the detection of CWD-prions was 30.77% in feces and 53.84% in blood. In contrast, in the less susceptible animals (PrP 96SS) the seeding activity was absent. These results further demonstrate the lower presence of CWD prions in excreta at early stages of the CWD incubation period.

### Influence of sex on CWD-prion detection using blood and feces

Finally, we evaluated if seeding activity was affected by the sex of the donor animal in both feces and blood. A fraction of 104 fecal samples from 88 female and 16 males, and seventy-three samples of blood (61 females and 12 males) were compared (Table 6). Our results showed a detection of PK-resistant PrP^Sc^ in fecal samples comparable between does (54.55%) and bucks (56.25%). Similar results were found when testing whole blood (females: 68.85%; males: 58.33%). These results suggest that the detection of CWD-prion seeding activity in feces and blood is not influenced by the deer’s sex.

**Table 6:** Influence of sex variation on CWD prion seeding activity considering different sample types from white-tailed deer.

Feces (n = 104)		Blood (n = 73)	
Female (n = 88) True positive rate* (detection%)	Male (n = 16) True positive rate* (detection%)	Female (n = 61) True positive rate* (detection%)	Male (n = 12) True positive rate* (detection%)
48/88* (54.55%)	9/16* (56.25%)	42/61* (68.85%)	7/12* (58.33%)

*True positive rate = n_PMCA positive_/n_IHC positive_.

## Discussion

CWD is continuously expanding in North America^71,72^, and it has been more recently reported in Northern Europe^4–6,73,74^. CWD is devastating socially and economically^75^, as there is no treatment or cure for this fatal disease and its zoonotic potential is still unknown. Significant practices have been implemented in surveilling this disease with the aim to reduce its spreading in both free-ranging and farmed populations. Additionally, considerable research efforts have been made towards the development of sensitive diagnostic tests^75^. A limitation of the currently accepted CWD diagnosis methods is their limited sensitivity and the fact that they need to be conducted *postmortem*^14,15,41,48^. Nonetheless, sensitive diagnostic methods such as RT-QuIC and PMCA have extensively demonstrated the detection of the infectious agent (i) in a wide range of biological and environmental samples^14,16–20,22,23,27,31,39,40,50,52,59,76–78^, (ii) long before the onset of clinical signs^14,37,39,40^, and (iii) in samples that harbor low amounts of CWD-prions. The main challenge for these tests is to optimize their use in biological samples suitable for *antemortem* collection. Candidate specimens for this include bodily fluids and excreta in which prions have been detected shortly post CWD prion exposure^37,39^. The purpose of this study was to compare the efficiency of PMCA to diagnose CWD in feces and blood. For that purpose, we considered multiple factors in white-tailed deer such as PrP polymorphic variability, prion incubation stages, and sex.

The first step to efficiently detect CWD prions using PMCA in feces was to optimize the best protocol for detection. PMCA inhibitors or competitors are present in multiple matrices as shown in multiple publications^35,52,55,56,67–69^. The use of NaPTA to enhance the detection of PrP^Sc^ has been well documented^14,15,37,39,50,66,67^. In our experience, the use of the ionic detergent sarkosyl and ultracentrifugation enhance prion detection by PMCA detection in multiple biological samples, including blood^56,57^, semen^58^, retropharyngeal and submandibular lymph nodes, and brain (Morales et al. unpublished). In our hands, the direct spiking of the feces homogenates into the PMCA substrate supports a higher efficiency in contrast to NaPTA or sarkosyl/ultracentrifugation pre-treatments. In fact, the lower seeding activity exhibited by both enrichment methods in different replicates was consistent in multiple assays performed in our laboratory (data not shown). One explanation for these unexpected observations is that some fecal-associated PMCA inhibitors co-precipitate with PrP^Sc^ particles, decreasing the efficiency of the cyclic amplification of misfolded prions. Another plausible explanation may be found in Colby et al.^79^ who described that NaPTA strongly inhibits amyloid formation whereas sarkosyl either inhibits or accelerates amyloid formation depending on its concentration in amyloid seeding assays. Although the protocols used in this study include washing steps to remove traces of NaPTA and sarkosyl, it is possible that certain components particularly pelleting in fecal samples co-precipitate with these reagents in quantities that are incompatible with PMCA. It is important to highlight that the blood and fecal specimens used in the present study were processed using different methods: sarkosyl/ultracentrifugation and direct spiking, respectively. As discussed above, the presence of inhibitors/competitors in different specimens may have varying effects in PMCA. The data presented in this study highlights the differential pre-PMCA processing steps needed for optimal detection in seeding assays, depending on the sample type. Nonetheless, more studies are necessary to accurately explain the PrP^Sc^ interaction with the feces biological matrix. Directly spiking PMCA reactions may be advantageous for diagnostic purposes as this substantially reduces the time and costs associated with the screening of a large number of samples. Importantly, testing of fecal samples may not only be relevant for animal diagnosis, but also for screening of premises where this sample type is abundant.

In regards to animal diagnosis, PMCA screening of fecal samples for this white-tailed deer cohort provided an overall 55.77% of accurate diagnosis. Other groups, have attempted to diagnose CWD in bodily fluids and excreta using prion seeding assays. Results on those studies varied depending on the sample type: 25% to 28% in urine^37,39^, 34% to 56% in nasal secretions^17,70^, 56% in semen^58^, 50% to 82% in saliva^35,37,55^, 71% to 79.5% blood^56,57^, and 60% to 83% in feces^16,39,40,61,68,69^. Considering this, our results are comparable to those found in other sample types. However, if we restrict our cohort to the most CWD-susceptible PrP 96 polymorphic group, and to late stages of the pre-clinical phase, the detection approaches to 100% as compared to results from the official *postmortem* IHC testing. Our data also suggest a higher diagnostic power of PMCA over RT-QuIC using feces considering the data of Tewari et al.^16^ who describe seeding activity in 70.5% of the animals that harbored PrP 96G alleles and CWD status positive in RPLN and brain by IHC. Although the diagnostic potential of feces appears to be low, they may still provide some utility to screen animals at their late CWD stages. The important drop in detection at early stages of the pre-clinical phase is a relevant issue for diagnostic purposes, and future studies should be devoted to optimize the PMCA protocol to increase sensitivity in this subset of samples. Regardless of these limitations, the use of fecal specimens for environmental screening could be an effective option for surveillance efforts in areas where CWD has not been detected. In addition, the use of fecal samples is advantageous since they are easily accessible and non-invasive to collect. In contrast, *postmortem* specimens are rare or inconvenient to obtain.

A relevant point in this screening involves the single false-positive result. Considering the consistent result in multiple replicates performed by different researchers, it is suggested that this sample was contaminated during the collection process. An alternative explanation is that this particular animal was at a very early stage of the disease and the accumulation of CWD prions within the lymph nodes was minimal and sparse (“patchy”). In addition, the fact that the animal was homozygous for serine at codon 96 of *PRNP* (a genotype that has been extensively described to be less susceptible to CWD) supports the assumption that low quantities of the infectious agent were present in this sample. Under this hypothetical scenario, this tissue may have displayed a patchy distribution of PrP^Sc^ and perhaps the portion used for IHC lacked PrP^Sc^ inclusions, making it histologically undetectable. However, it is important to note that blood from this animal lacked seeding activity or was under the PMCA threshold. Considering the PMCA data collected from the feces and blood from this animal, the most plausible explanation is that the fecal matter was contaminated during the collection process.

Different studies demonstrate that the genetic variability at the codon 96 of the white-tailed deer prion protein has a strong effect on both, the prevalence of CWD and the incubation period^13,14,33,80,81^. Likewise, the accumulation of PrP^Sc^ in different tissues across the course of the disease appears to be modulated by these polymorphic variations, affecting CWD detection^13,14,19,24,42,82^. In this study, we compared the PMCA seeding activity in feces and blood from white-tailed deer harboring different PrP 96 polymorphic variants and at different stages of the pre-clinical phase. Our results demonstrated that (i) CWD-prions are more available for detection in feces and blood at late stages of the pre-clinical phase, (ii) the PrP 96G allele favors detection in both sample types in a dose dependent manner, and (iii) at the early pre-clinical stage blood provides a better diagnostic power compared with feces. Considering that direct spiking of feces homogenates was the most efficient mean to detect prions using PMCA, it is suggested that the feces matrix contain less PMCA inhibitors compared with blood (sample that requires a sarkosyl/ultracentrifugation pre-treatment for PMCA detection). Along the same line, the data presented in this study suggests that feces contain lower quantities of PrP^Sc^ compared with blood. Alternatively, blood may harbor higher CWD-prion titers at late stages due to its role in the trafficking of the infectious agent from and to diverse peripheral tissues.

Our results also demonstrate that the overall presence of CWD in farmed white-tailed deer is not influenced by sex. This is different compared to free-ranging deer population where CWD incidence seems to be higher in males. These contradictory results may be due to the confinement experienced in farming settings. In addition, we acknowledge that our deer cohort includes a substantially higher number of females over males, fact that may have influenced these results. The analysis of larger and sex-equivalent deer cohorts from free-ranging and farmed deer may help to elucidate properly the effect of sex in CWD incidence, prevalence and diagnosis.

## Conclusions

In summary, here we report the dynamics of CWD prion detection in fecal and blood specimens across the pre-clinical phase of infection. CWD-prions were best detected in deer carrying both copies of the PrP 96G codon, particularly during the late pre-clinical stage, in both fecal and blood samples. In addition, our results demonstrate that blood exhibited a higher diagnostic potential before neuroinvasion occur in infected deer. Overall, our findings contribute to the development of prion diagnosis methodologies and to the understanding of the prion distribution and burden across different biological samples during the course of the disease in naturally-infected, farmed deer.

### Supplementary Information

Supplementary Information.

## Data Availability

All data generated or analyzed during this study are included in this published article and Supplementary Information. Raw data are available upon reasonable request to the corresponding author.

## Abbreviations

(w/v): (Weight/volume)
96G: Glycine at position 96 of the prion protein
96GG: Homozygous for glycine at position 96 of the prion protein
96GS: Heterozygous for glycine and serine at position 96 of the prion protein
96SS: Homozygous for serine at position 96 of the prion protein
CWD: Chronic wasting disease
EDTA: Ethylenediaminetetraacetic acid
ELISAs: Enzyme-linked immunosorbent assays
IHC: Immunohistochemistry
MRLPN: Medial retropharyngeal lymph node
NaPTA: Sodium phosphotungstic acid
PBS: Phosphate-buffered saline
PK: Proteinase K
PMCA: Protein misfolded cyclic amplification
*PRNP*: Prion protein gene
PrP: Prion protein
PrP^C^: Host cellular prion protein
PrP^Sc^: Infectious prion protein
RAMALT: Recto anal mucosa-associated lymphoid tissue
RPLN: Retropharyngeal lymph node
RT-QuIC: Real-time quaking-induced conversion
SDS: Sodium dodecyl sulfate
USDA: U.S. Department of Agriculture
UTHealth: The University of Texas Health Science Center at Houston
