# Supplementary material for: Dynamics of CWD prion detection in feces and blood from naturally infected white-tailed deer
Source: Sci Rep. 2023 Nov 17;13:20170. doi: 10.1038/s41598-023-46929-9 (PMC10656452; doi:10.1038/s41598-023-46929-9)
Supplement: Supplementary file 1 — Supplementary Information. [file 41598_2023_46929_MOESM1_ESM.pptx]

## Slide 1
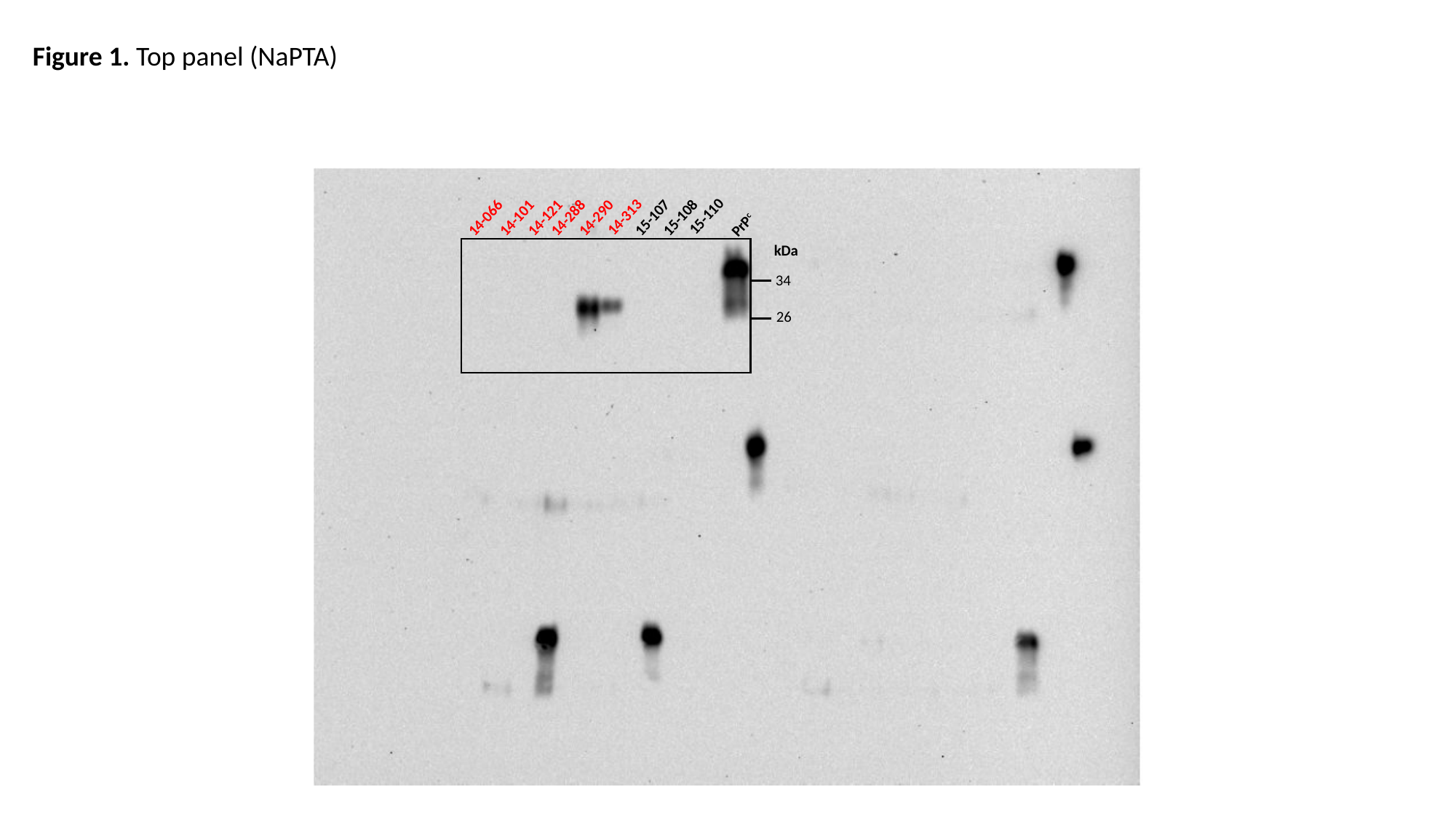

Figure 1. Top panel (NaPTA)
15-110
14-313
15-108
14-066
14-101
14-290
14-121
14-288
15-107
PrPc
kDa
34
26

## Slide 2
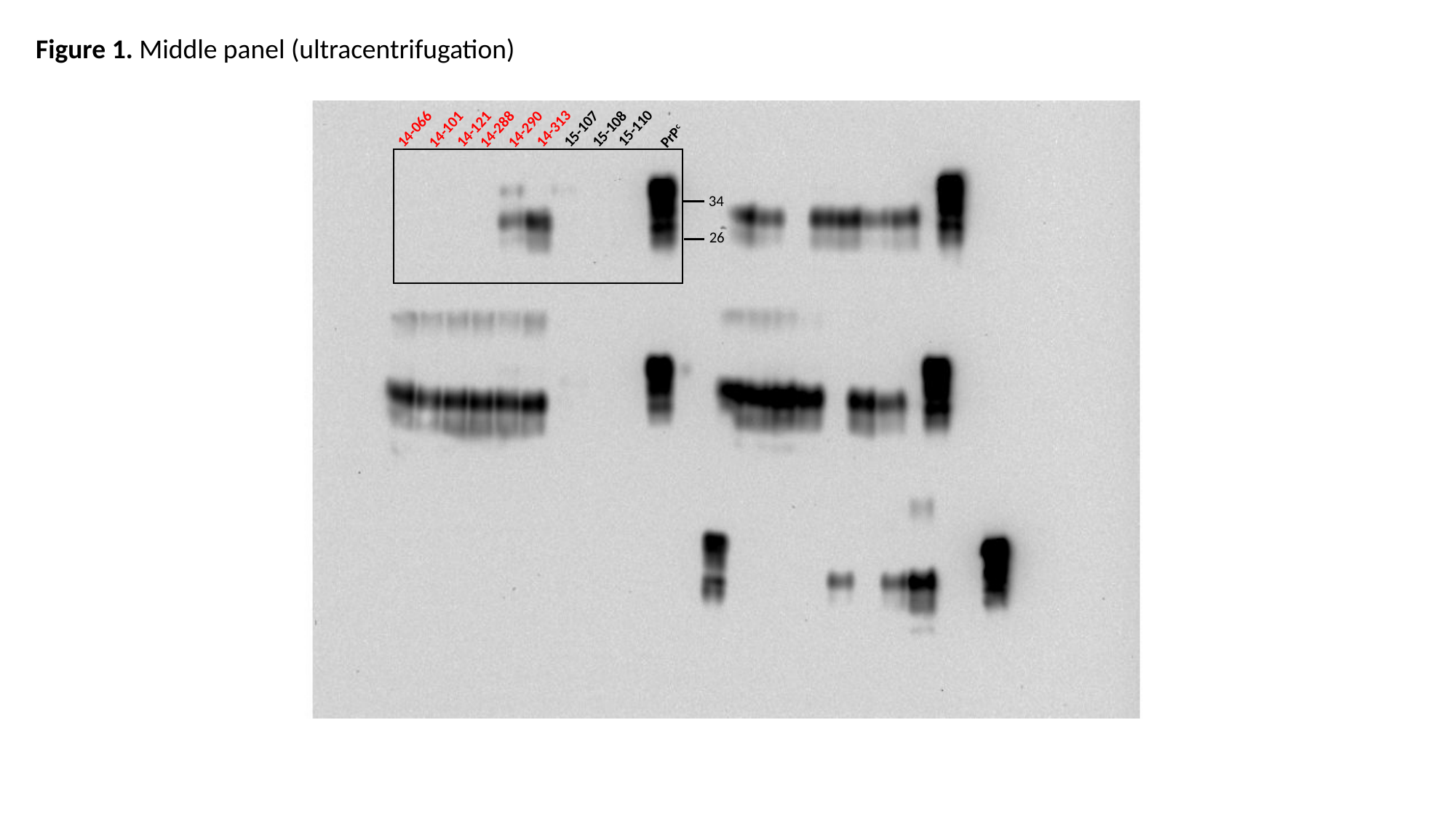

Figure 1. Middle panel (ultracentrifugation)
15-110
14-313
15-108
14-066
14-101
14-290
14-121
14-288
15-107
PrPc
34
26

## Slide 3
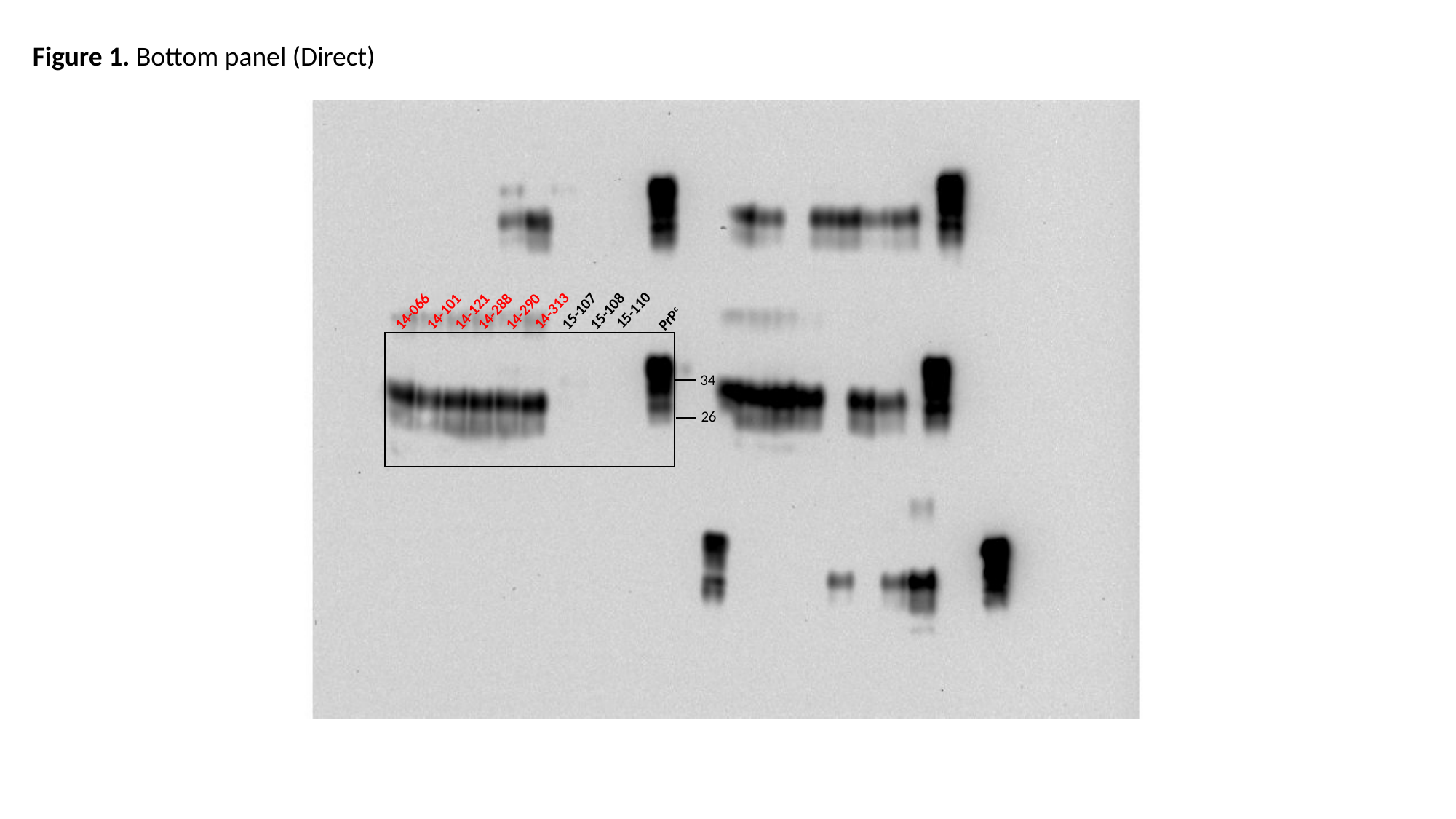

Figure 1. Bottom panel (Direct)
15-110
14-313
15-108
14-066
14-101
14-290
14-121
14-288
15-107
PrPc
34
26
